# Real-world correlation between therapeutic drug monitoring and clinical outcomes of the antifungal drug posaconazole: a single-center retrospective cohort study

**DOI:** 10.3389/fphar.2025.1592767

**Published:** 2025-09-10

**Authors:** Qiaoyan Yi, Lin Dong, Peng Wang, Yinping Shi, Yilei Yang, Zhonghua Dong, Siwen Li, Yan Li, Xin Huang, Jinjuan Liu, Haiyan Shi, Hongmei Wang

**Affiliations:** ^1^ Department of Clinical Pharmacy, The First Affiliated Hospital of Shandong First Medical University and Shandong Provincial Qianfoshan Hospital, Shandong Engineering and Technology Research Center for Pediatric Drug Development, Jinan, Shandong, China; ^2^ Department of Hematology, The First Affiliated Hospital of Shandong First Medical University and Shandong Provincial Qianfoshan Hospital, Jinan, Shandong, China; ^3^ Department of Pediatrics, The First Affiliated Hospital of Shandong First Medical University and Shandong Provincial Qianfoshan Hospital, Jinan, Shandong, China

**Keywords:** posaconazole, therapeutic drug monitoring, invasive fungal disease, prophylactic antifungal therapy, hematopoietic stem cell transplantation

## Abstract

**Objective:**

Posaconazole is a first-line drug for preventing invasive fungal disease (IFD) in patients undergoing haematopoietic stem cell transplantation (HSCT). Few retrospective studies have examined the impact of therapeutic drug monitoring (TDM) on preventing IFD with posaconazole. This study was designed to evaluate the efficacy, safety, and cost-effectiveness of posaconazole in preventing IFD based on real-world data.

**Methods:**

This single-centre, retrospective cohort study analyzed the use of posaconazole for fungal prophylaxis in HSCT patients at the First Affiliated Hospital of Shandong First Medical University (Shandong Provincial Qianfoshan Hospital). Patients were classified into TDM and non-TDM groups based on their TDM status. Clinical data were analyzed using propensity score matching (PSM) to further elucidate the role of TDM in posaconazole prophylaxis.

**Results:**

After PSM, the prophylactic success rate was significantly higher in the TDM group (100%) than in the non-TDM group (52.9%) (P = 0.003). There was no statistically significant difference in gastrointestinal, hepatic and renal adverse effects between the two groups (P > 0.05). Both the total cost of treatment and the cost of medication were lower in the TDM group compared to the non-TDM group.

**Conclusion:**

Real-world data demonstrate that TDM enhances the effectiveness of posaconazole in preventing IFD in HSCT patients and moderately reduces treatment costs.

## 1 Introduction

With the increasing prevalence of immunodeficiency and critically ill patient populations, the incidence of invasive fungal disease (IFD) is rising annually. The proportion of fungal infections, such as *Candida* albicans, Aspergillus, and Trichophyton rubrum, is also increasing among hematological patients. Haematopoietic stem cell transplantation (HSCT) remains the sole curative treatment for a broad spectrum of hematological and other systemic malignant diseases. IFD represents one of the most serious infectious complications and a primary cause of mortality post-HSCT. Factors such as prolonged neutropenia, suboptimal engraftment, and immunosuppressive therapy during the transplantation process markedly elevate the risk of IFD (Jenks et al., 2020). The preventive administration of antifungal drugs has significantly diminished the incidence of IFD and potential IFD-related mortalities (Marr et al., 2000; Gao et al., 2016). Posaconazole, as a first-line prophylactic agent, exhibits distinct advantages. As a broad-spectrum triazole antifungal, it not only possesses potent antifungal properties but also effectively prevents various fungal infections, including *Candida*, Aspergillus, Coccidioides, and Histoplasma (Barchiesi et al., 2000; Girmenia, 2009). It functions by inhibiting ergosterol synthesis via blockade of the cytochrome P450-dependent 14α-demethylase, which disrupts biosynthesis and alters membrane permeability, thereby inhibiting fungal proliferation (Lass-Flörl, 2011). Owing to its clinical efficacy and tolerability, both domestic and international guidelines strongly recommend posaconazole as a first-line prophylactic treatment for IFD (Patterson et al., 2016; Maertens et al., 2018), and its use is widespread in clinical settings.

As a second-generation triazole, posaconazole offers a broader antifungal spectrum, high bioavailability, minimal drug resistance, and fewer drug interactions (Kwon and Mylonakis, 2007; Moore et al., 2015). It is primarily metabolized through the glucuronidation pathway and predominantly inhibits the activity of CYP3A enzymes, sparing CYP2C19, and thus presents a lower risk of drug interactions (Xiao-Qun et al., 2011). Significant variability is observed in the pharmacokinetics of posaconazole among individuals. The absorption rate constant (Ka) and bioavailability (F) of posaconazole exhibit considerable variability (Dolton et al., 2014; Chen et al., 2020). Approximately 17% of its metabolism occurs via uridine diphosphate (UDP)-glucuronosyltransferase, and inducers of this enzyme, such as phenytoin and rifabutin, can reduce plasma concentrations of posaconazole (Shu et al., 2022). Studies have linked plasma concentrations of posaconazole directly to its clinical efficacy (Jang et al., 2010; Dolton et al., 2012), with the target minimum plasma concentration (Cmin) for preventing IFD established at no less than 0.7 μg/mL (Jang et al., 2010). However, due to pronounced pharmacokinetic diversity, some patients may not achieve the desired prophylactic effect due to suboptimal plasma concentrations (Van der Elst et al., 2015). Therefore, therapeutic drug monitoring (TDM) is crucial for patients on posaconazole to ensure drug efficacy and minimize resistance.

TDM is a clinical pharmacy service that ensures drug concentrations are maintained within an effective treatment range through quantitative analysis of patient drug levels. It involves adjusting drug dosages to enhance treatment efficacy and minimize side effects. With TDM, physicians can continuously monitor a patient’s drug concentrations, ensuring the drug remains within the therapeutic window and enhancing treatment outcomes. For patients using posaconazole to prevent IFD, TDM can promptly detect the status of plasma concentrations. Therefore, TDM is recommended for antifungal prophylaxis with posaconazole (John et al., 2019).

Despite these recommendations, the published literature lacks comprehensive controlled studies on the effectiveness, safety, and cost-effectiveness of TDM in the Chinese population, especially in the context of prophylactic use of posaconazole. Consequently, our study retrospectively evaluated the impact of TDM on the efficacy, safety, and cost-effectiveness of posaconazole by examining HSCT patients who used posaconazole for fungal prophylaxis. Our research aims to provide a reference for the rational use of posaconazole in clinical practice, aiming to optimize fungal prophylaxis regimens and improve treatment outcomes and survival rates for patients.

## 2 Materials and methods

### 2.1 Ethical approval

The study was designed to adhere to legal requirements and the Declaration of Helsinki. Ethical approval was granted by the Ethics Committee of Shandong First Medical University (approval number R526). All methods were conducted in accordance with the relevant guidelines. Due to its retrospective nature, the institution waived the requirement for obtaining written informed consent from patients.

### 2.2 Study design and participants

This retrospective, single-center study enrolled HSCT patients who used posaconazole oral suspension or enteric-coated tablets for IFD prevention and underwent TDM from January 2021 to March 2024 at the First Affiliated Hospital of Shandong First Medical University.

This study was divided into two groups: The TDM group: patients who received posaconazole TDM; Non-TDM group: Patients who did not undergo posaconazole TDM.

Inclusion criteria: (1) Patients with hematological diseases undergoing HSCT; (2) Prophylaxis with posaconazole oral suspension or enteric-coated tablets; (3) TDM group: the steady state plasma concentration (≥7 days) must be monitored; Non-TDM group: Plasma concentrations must not have been monitored. The exclusion criteria were: (1) Incomplete data or medication information; (2) Active fungal infection; (3) Non-adherence to medical advice; (4) Patients in the TDM group whose steady-state trough concentration (≥7 days of administration) was not recorded.

Data collection included: (1) Demographic data, including age, gender, and body weight; (2) Hepatic and kidney function tests, including gamma-glutamyl transferase (GGT), alanine aminotransferase (ALT), aspartate aminotransferase (AST), total bilirubin (TBIL), and blood urea nitrogen (BUN); (3) Posaconazole-specific data, such as usage and dosage, sampling times, and minimum concentration (Cmin) results; (4) Concomitant medications, including proton pump inhibitors (PPI) and phenytoin; (5) Pathological symptoms reported by patients, such as nausea, vomiting, and diarrhea. These data were compiled into a database.

### 2.3 Drugs and reagents

Posaconazole oral suspension (Specification: 40 mg·mL-1, Trade name: Noxafil^®^); Posaconazole enteric-coated tablets (Specification: 100 mg, Trade name: Noxafil^®^); Tinidazole internal standard working solution (Concentration: 250 μg/mL); formic acid and acetonitrile were both of chromatographic grade.

### 2.4 Sample collection and bioanalysis

Posaconazole TDM trough concentrations were monitored. Blood samples were collected 0.5 h before the first dose of the day. The concentration of the sample collected before the first dose was defined as the C_min._

The target trough C_min_ for posaconazole is 0.7 μg/mL. After 7 days of administration, serum samples containing posaconazole were obtained from patients using EDTA tubes. Posaconazole blood concentrations were determined by HPLC. The methodology conformed to the standards set in the 2020 edition of the Chinese Pharmacopoeia (Volume IV) and the 2018 FDA Guidance on Bioanalytical Technique Validation. Only one blood concentration measurement per patient was included in the analysis.

### 2.5 Evaluation indicators

The clinical diagnosis of IFD was defined as at least one host factor, one clinical criterion and one microbiological criterion (Chinese Association HematologistsChinese Invasive Fungal Infection Working Group, 2020).

Host factors included: (1) recent neutropenia (neutrophil count <500/L) lasting for more than 10 days; (2) receiving allogeneic hematopoietic stem cell transplantation; (3) use of glucocorticoids (more than 0.3 mg kg d, except allergic bronchopulmonary aspergillosis) for more than 3 weeks within the past 60 days; (4) Use of T cell immunosuppressants (such as cyclosporine A, tumor necrosis factor, some monoclonal antibodies such as alemtuzumab) or nucleoside analogues within 90 days; (5) use of B cell immunosuppressive agents such as BTK inhibitors; (6) history of invasive fungal infection; (7) patients with AIDS or genetic immunodeficiency (such as chronic granulomatosis or combined immunodeficiency).

Clinical criteria included: (1) For lower respiratory tract fungi, the presence of at least one of the following four items on CT examination: dense, well-circumscribed lesion (with or without halo sign), air crescent sign, cavity, wedge/segmental or lobar lesion; Other filamentous fungi include: reverse halo sign; (2) For tracheobronchitis, bronchoscopy showed the following manifestations: tracheobronchial ulcers, nodules, pseudomembranes, plaques or crusts; (3) For sinus infection, at least one of the following: acute local pain (including pain radiating to the eyes), nasal ulcers with black crusts, erosion of bone from the sinuses, including intracranial spread; (4) For central nervous system: at least one of the following: imaging examination suggested focal lesions; MRICT examination showed meningeal enhancement. For disseminated candidiasis, candidemia with at least one of the following within the previous 2 weeks: hepatic/spleen bull’s eye sign; An ophthalmic examination revealed progressive retinal exudation.

#### 2.5.1 Microbiological criteria include

Direct examination: cytology, direct microscopic examination or culture: (1) The presence of at least one of the following in sputum, bronchoalveolar lavage fluid, bronchial brush or sinus aspirate indicated mold infection: the presence of fungal components showed mold, culture indicated mold; (2) Sputum or bronchoalveolar lavage fluid was positive for Cryptococcus neoformans culture or was found by direct microscopic or cytological examination.

Indirect examination: detection of antigens or cell wall components. (1) Galactomannan test (GM test) positive serum (1,3) -B-D-glucan test (G test) positive for Aspergillus in plasma, serum, bronchoalveolar lavage fluid or cerebrospinal fluid; (2) For invasive fungal diseases (except cryptococcosis and zygomycosis): serum (1,3) -B-D-glucan test (G test) was positive; (3) For Cryptococcus, Cryptococcus capsular polysaccharide antigen was positive.

Clinical efficacy indicators were categorized as effective and ineffective prophylaxis. Effective prophylaxis was defined as the absence of fungal infection from the start of posaconazole treatment until 3 months later. Posaconazole was considered ineffective if a breakthrough fungal infection occurred during treatment or within 3 months after the use of the drug.

The safety evaluation focused on gastrointestinal disturbances (including abdominal distension, diarrhea, nausea and vomiting) as well as hepatic and renal adverse reactions, assessed according to the Common Terminology Criteria for Adverse Events (CTCAE) (Xu et al., 2017). Among them, hepatic function and kidney function were converted into categorical variables: normal and abnormal. Hepatic function was considered normal if ALT levels were ≤2ULN (ULN: 50 U/L), AST levels were ≤2ULN (ULN: 40 U/L), and TBIL levels were ≤24 μmol/L. Renal function indicators were converted into categorical variables: Normal/Abnormal. Renal function was considered normal if the patient’s creatinine clearance rate (Ccr) was ≥60 mL/min/1.73 m^2^.

The economic evaluation compared incremental cost-effectiveness ratios between the two groups. Total treatment cost was calculated as the total hospitalization cost for patients. Drug cost referred to the medication cost. Drug cost ratio was the ratio of drug cost to total treatment cost. The change in efficacy (ΔE) was the difference between the TDM group’s prophylaxis success rate and the non-TDM group’s rate. The incremental cost-effectiveness ratio (ICER) for total treatment cost and drug cost was calculated as the change in cost divided by the change in efficacy.

### 2.6 Statistical analysis

To minimize selection bias between the TDM and non-TDM groups, propensity score matching (PSM) was performed for a total of 56 patients from both groups. Patients were matched 1:1 using the nearest neighbour method with a caliper value of 0.3. Propensity scores were computed based on gender, BMI, gastrointestinal function, hepatic function, kidney function and concomitant medication. Ultimately, 17 matching pairs were formed in each of the TDM group and the non-TDM group (n = 34).

Continuous variables were presented as mean ± SD or median (IQR) and categorical variables as numbers and percentages. Differences in baseline characteristics between the TDM and non-TDM groups were examined using t-tests or Mann–Whitney U tests for continuous variables and Pearson’s χ^2^ test or Fisher’s exact test for categorical variables. The significance level was set at p < 0.05. All statistical tests were conducted using SPSS version 22.0 software (SPSS Inc.).

## 3 Results

### 3.1 Patient information

According to the inclusion criteria, a total of 44 patients were included in the TDM group and 30 patients were included in the non-TDM group. According to the exclusion criteria, in the TDM group, 4 cases were excluded due to the presence of active fungal infections during the baseline period, 4 cases were excluded due to the lack of steady-state concentration data, and 4 cases were excluded due to incomplete data information. In the non-TDM group, 2 cases were excluded due to active fungal infections during the baseline period, and 2 cases were excluded due to incomplete data and information. Therefore, there were ultimately 30 patients in the TDM group and 26 patients in the non-TDM group.

**FIGURE 1:**
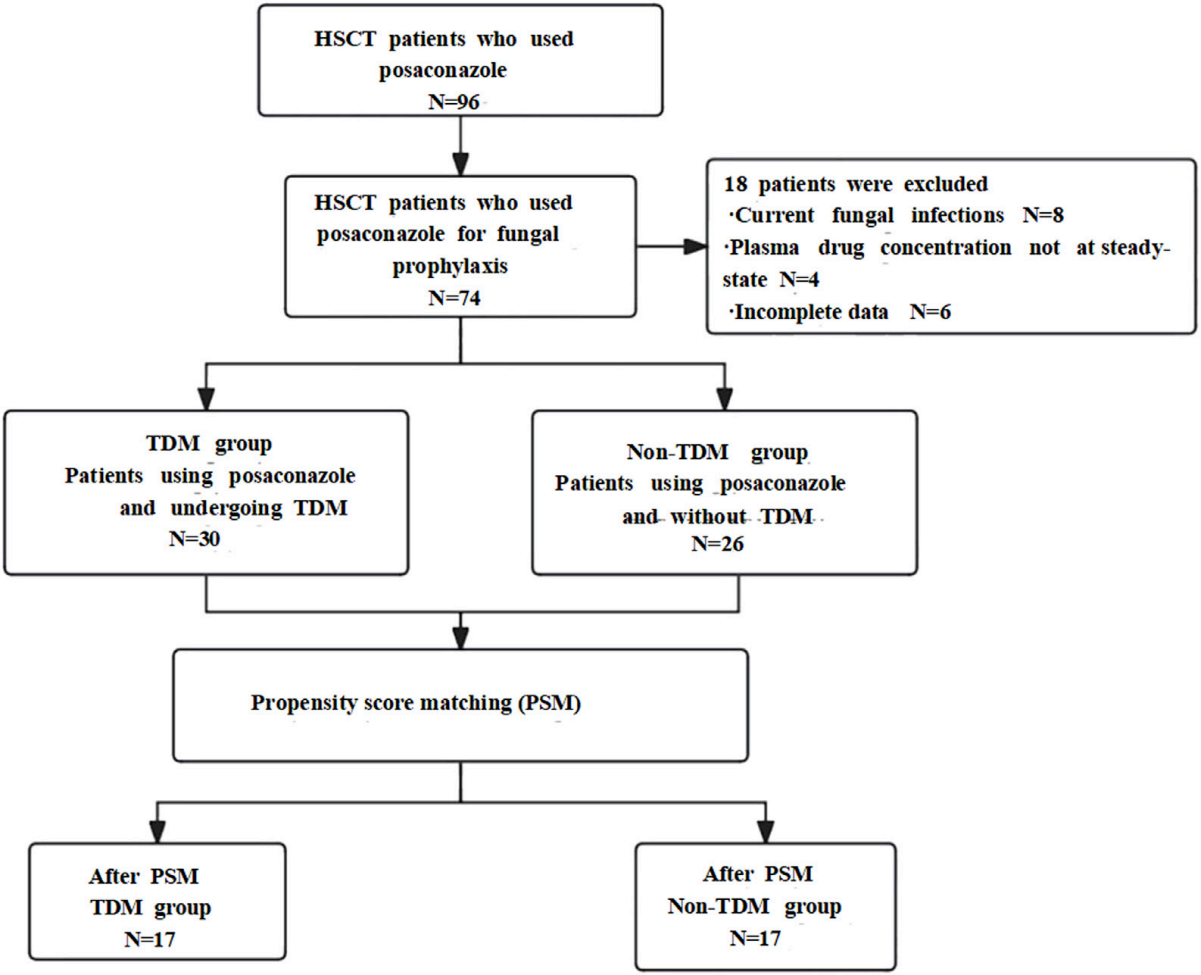
Flowchart of case selection and results.

The baseline characteristics of the included patients are shown in Table 1. Prior to matching, there were 30 patients in the TDM group and 26 in the non-TDM group. No statistical differences were observed between the groups in terms of gender, BMI, albumin levels, NEUT, gastrointestinal functions, kidney functions, and concomitant medication (Omeprazole, phenytoin, metoclopramide, Cyclosporin/Tacrolimus) (P > 0.05). However, significant difference was noted in hepatic function (P < 0.05). After PSM, the TDM group comprised 17 patients and the non-TDM group 17 patients. Post-PSM, no statistical differences were noted between the groups in any variables (P > 0.05), indicating that baseline disparities had been addressed, rendering the groups comparable. All patients had no active fungal infection at the baseline period. The specific inclusion process is shown in Figure 1.

**TABLE 1:** Clinical feature between the two groups before and after propensity score matching.

Features	All patients			Propensity-matched patients		
	TDM (n = 30)	Non-TDM (n = 26)	P value	TDM (n = 17)	Non-TDM (n = 17)	P value
Gender, n (%)			0.206			0.732
Men, n (%)	20 (66.7)	13 (50.0)		10 (58.82)	8 (47.06)	
Women, n (%)	10 (33.3)	13 (50.0)		7 (41.18)	9 (52.94)	
BMI(IQR)	15.84 (14.68,17.86)	15.91 (14.93,17.86)	0.339	16.97 (14.80,19.08)	15.98 (15.07,17.49)	0.384
Albumin, g/L, median (IQR)	40.70 (36.08,45.03)	42.70 (39.75,46.15)	0.104	40.70 (36.10,44.00)	43.50 (39.30,46.40)	0.233
NEUT, ×10^9^cells/L, median (IQR)	1.27 (0.04,2.69)	2.48 (0.34,2.48)	0.111	1.05 (0.025,2.12)	0.50 (0.15,3.09)	0.863
Liver^a^, n (%)			0.002			>0.999
Normal	21 (70.0)	26 (100.0)		16 (94.1)	17 (100)	
Abnormal	9 (30.0)	0 (0)		1 (5.9)	0 (0)	
Gastrointestinal^b^, n (%)			0.838			>0.999
Normal	20 (66.7)	18 (69.2)		10 (58.82)	9 (52.94)	
Abnormal	10 (33.3)	8 (30.8)		7 (47.06)	8 (47.06)	
Kidney^c^, n (%)			0.464			NA
Normal	30 (100)	25 (96.2)		17 (100)	17 (100)	
Abnormal	0 (0)	1 (3.8)		0 (0)	0 (0)	
Concomitant medications, n (%)						
Omeprazole			0.757			>0.999
Yes	7 (23.3)	7 (26.9)		4 (23.5)	4 (23.5)	
No	23 (76.7)	19 (73.1)		13 (76.5)	13 (76.5)	
Phenytoin			>0.999			>0.999
Yes	2 (6.7)	2 (7.7)		2 (11.8)	2 (11.8)	
No	28 (93.3)	24 (92.3)		15 (88.2)	15 (88.2)	
Metoclopramide			>0.999			NA
Yes	1 (3.3)	0 (0)		0 (0)	0 (0)	
No	29 (96.7)	26 (100)		17 (100)	17 (100)	
Cyclosporin/Tacrolimus			0.158			0.485
Yes	26 (96.7)	26 (100)		15 (88.2)	17 (100)	
No	4 (3.3)	0 (0)		2 (11.8)	0 (0)	

^a^ Hepatic function was considered normal if ALT, levels were ≤2ULN(ULN: 50 U/L), AST, levels were ≤2ULN(ULN: 40 U/L), and TBIL, levels were ≤24 μmol/L.

^b^ Gastrointestinal function was considered normal if the patient did not experience any of the following: nausea, vomiting or diarrhea.

^c^ Renal function was considered normal if the patient’s creatinine clearance rate (Ccr) was ≥60 mL/min/1.73 m2.

NA: statistical testing was not performed because the outcome had no variability.

### 3.2 Comparison of prophylactic effectiveness after matching

After PSM, 17 patients (100%) in the TDM group achieved successful prophylaxis. In the non-TDM group, 9 patients (52.9%) were successful, and 8 patients (47.1%) failed. The chi-square test indicated a statistically significant difference between the two groups (P = 0.003), as shown in Table 2. In the non-TDM group, 6 out of 8 patients who failed in prophylaxis had Aspergillus infection, and both G test and GM test were positive. In the other 2 cases, only G test was positive and the fungal species could not be determined.

**TABLE 2:** Comparison of clinical outcomes between the two groups after propensity score matching analysis.

Group	Clinical outcomes	
	Success	Failure
TDM, n (%)	17 (100)	0 (0)
Non-TDM, n (%)	9 (52.9)	8 (47.1)
χ^2^ value	10.462	
P value	0.003	

### 3.3 Safety analysis after PSM

Adverse events occurred in 7 patients in the TDM group (41.2%). Among them, 2 patients had gastrointestinal adverse reactions and 5 patients had abnormal liver function. Adverse reactions occurred in 6 patients in the non-TDM group (35.3%). There were 1 case of rash, 3 cases of gastrointestinal adverse reactions and abnormal liver function, and 2 cases of abnormal liver function. No statistically significant differences were found in the rates of gastrointestinal and liver adverse events between the groups (P > 0.05), though a increase in the incidence of adverse events was observed in TDM group, as depicted in Table 3. No adverse events related to the kidneys and cardiovascular system occurred in either group of patients.

**TABLE 3:** Comparison of adverse events rates between the two groups.

Group	Skin function^a^		Gastrointestinal funtion^b^		Hepatic function^c^		Total	
	Normal	Abnormal	Normal	Abnormal	Normal	Abnormal	Normal	Abnormal
TDM, n (%)	17 (100.0)	0 (0.0)	15 (88.2)	2 (11.8)	12 (70.6)	5 (29.4)	10 (58.8)	7 (41.1)
Non-TDM, n (%)	16 (94.1)	1 (5.9)	14 (82.4)	3 (17.6)	12 (70.6)	5 (29.4)	11 (64.7)	6 (35.3)
χ^2^ value	1.030		0.234		0.151		0.515	
P value	>0.999		>0.999		>0.999		0.721	

^a^ Skin disorders mainly refer to rashes.

^b^ Gastrointestinal function indicators were converted into categorical variables: Normal/Abnormal. Gastrointestinal function was considered normal if the patient did not experience any of the following: nausea, vomiting or diarrhea.

^c^ Hepatic function indicators were converted into categorical variables: Normal/Abnormal. Hepatic function was considered normal if ALT, levels were ≤2ULN(ULN: 50 U/L), AST, levels were ≤2ULN(ULN: 40 U/L), and TBIL, levels were ≤24 μmol/L.

### 3.4 Cost-effectiveness analysis

Incremental cost-effectiveness ratios (ICER) were used to compare the medical costs of the TDM and non-TDM groups. The results showed that in the TDM group, the *per capita* total cost was reduced by 24524.82 yuan, the *per capita* total drug cost was reduced by 24123.29 yuan, and the compliance rate was increased by 47.1%. ICER can save 520.70 yuan of hospitalization cost and 512.17 yuan of drug cost for every 1% increase in the target rate, which has absolute advantages (Table 4). Sensitivity analysis confirmed that even if the test cost increased by 10%, the ICER(total cost) was −97.05 yuan, which still maintained the economic advantage and supported the clinical promotion of TDM.

**TABLE 4:** Incremental cost-effectiveness analysis between the two groups.

Group	Average total cost (¥)	Average total drug cost (¥)	Drug cost ratio	Effective rate	ICER (total cost)	ICER (total drug cost)
TDM	199540.01	111346.26	49.89%	100%	−520.70	−512.17
Non-TDM	224064.83	135469.55	58.45%	52.9%		
P value	0.616	0.459				

## 4 Discussion

Invasive fungal infections have emerged as a leading cause of mortality among HSCT patients, significantly affecting transplantation outcomes and long-term patient prognosis (Busca and Pagano, 2016; Li et al., 2017; Otto and Green, 2020). With the use of prophylactic antifungal drugs and immunosuppressive agents, the epidemiology of these infections has shifted. The prevalence of *Candida* infections has declined, while that of Aspergillus infections has risen (Schmiedel and Zimmerli, 2016; Souza et al., 2021). Thus, when selecting prophylactic antifungal medications, consideration of various potential drug-resistant strains is necessary. This study was retrospective and included patients with baseline biases, which were mitigated by employing PSM to reduce selection bias. The PSM method ensures comparability of baseline data between groups, enhancing the reliability of the results.

PASS software (15.0.5) was used to calculate the sample size of the two groups, and the results showed that with the effective rate of 100% in the TDM group and 52.9% in the non-TDM group (Target power set to 0.9, alpha set to 0.05), the sample size of each group should be at least 12 cases. In this study, there were 17 cases in the TDM group and 17 cases in the non-TDM group after PSM, which met the sample size requirements.

The study revealed a prevention success rate of 100% in the TDM group, compared to 52.9% in the non-TDM group. The clinical prophylactic efficacy of the TDM group was significantly greater than that of the non-TDM group (P = 0.003). This suggests that TDM can enhance the clinical efficacy of posaconazole. Thus, based on TDM results, the posaconazole dosing regimen may be adjusted to maintain drug concentrations within the target range, optimizing efficacy and minimizing toxicity. This finding aligns with previous studies (Lewis et al., 2019), which have shown that hospitals implementing TDM for posaconazole report superior treatment outcomes.

In the TDM group, 8 of 17 patients achieved the target prophylactic concentration of posaconazole (≥0.7ug/mL). In order to prevent adverse events and save drug costs, the dose of 300 mg qd was adjusted to 100 mg qd. The prevention of this child was effective, and no adverse events occurred. Among the 9 cases whose initial concentration was below the target, 3 cases had the dose increased, and 6 cases with very low concentration were adjusted to other antifungal drugs. All children in the TDM group were successfully prevented from fungal infection.

Posaconazole is highly safe, with an adverse reaction rate lower than that of voriconazole (Cornely et al., 2007). The principal adverse events to posaconazole are hepatic and gastrointestinal function abnormalities, which are typically mild, with serious adverse event rates ranging from 6% to 13% (Cornely et al., 2007; Ullmann and Jeffrey, 2007; Winston et al., 2011). To date, no adverse events directly related to posaconazole drug concentrations have been reported, making discontinuation due to adverse effects uncommon (Dolton et al., 2014). This study investigated the effect of hepatic enzyme inducer (phenytoin) and P-glycoprotein inhibitor (omeprazole, cyclosporine) on the plasma concentration of posaconazole and its clinical significance. The results showed that phenytoin as a UDP-glucosidase inducer significantly reduced posaconazole concentrations (<0.7 μg/mL in both patients with combination therapy), which was consistent with expectations and suggested strict monitoring during clinical combination therapy. The effect of P-gp inhibitors was bidirectional: although omeprazole may increase intestinal absorption through P-gp inhibition, its inhibition of gastric acid was dominant. Only 1 case of 4 cases combined with omeprazole reached the standard concentration (≥0.7 μg/mL). Cyclosporine as a P-gp inhibitor can theoretically increase the concentration of P-gp. In this study, the concentration of 8 patients in the TDM group reached the target rate of 50%. Of note, although these drug interactions significantly affected posaconazole exposure, there was no statistically significant difference in the incidence of adverse events between the two groups, possibly because of: (1) the small sample size made it difficult to detect differences; (2) The fluctuation of posaconazole concentration did not reach the threshold of significantly affecting the safety; (3) The non-TDM group may avoid extreme high concentration due to empirical medication. TDM was of great value in this study, identifying not only the risk of undertreatment (sub-target concentration) in PHT co-users, but also potential overdose cases (4.58 μg/mL) in omeprazole co-users, suggesting that TDM can optimize individualized dosing and balance efficacy and safety. Therefore, TDM is recommended for patients combined with hepatic enzyme inducers or P-gp inhibitors to avoid treatment failure or drug toxicity, especially in high-risk groups.

In this study, treatment costs in the TDM group were significantly lower than those in the non-TDM group. Cost-effectiveness analysis indicated that the TDM group offered better economic value. For every unit increase in efficacy, total treatment costs for patients in the TDM group were 520.70 yuan lower than for those in the non-TDM group, and drug costs were 512.17 yuan lower. From the perspective of pharmacokinetic/pharmacodynamic (PK/PD) optimization, TDM achieves cost control at multiple levels by precisely adjusting the administration schedule of posaconazole. First, by avoiding the “trial and error” process of empirical administration, TDM significantly reduces the costs of subsequent rescue therapy and adverse effect management by reducing treatment failure due to insufficient dose (e.g., breakthrough fungal infection) or toxic effects caused by overdose (e.g., liver injury). The lower drug costs in the TDM group in this study reflect that PK/PD-guided dose optimization (e.g., timely dose escalation for phenytoin users and dosage modification for omeprazole users) can reduce exposure to ineffective drugs. Second, TDM shortened the time window to reach the target concentration, and avoided prolonged hospitalization caused by delayed efficacy by early identification of patients with low concentration (such as 2 phenytoin users), which explained the economic advantage of a total treatment cost reduction of 520.70 yuan per 1% improvement in efficacy. More important, concentration-based prophylaxis (e.g., in patients undergoing hematopoietic stem-cell transplantation) can reduce the incidence of invasive fungal disease and essentially circumvent costly rescue therapies. These mechanisms together show that the economic value of TDM is not only reflected in the savings in direct drug costs, but also from the improvement in the overall efficiency of diagnosis and treatment brought by PK/PD optimization, which provides clinicians with both accurate and cost-effective decision-making tools.

This study also has limitations: on one hand, PSM can only balance known impact factors and cannot adjust for unknown factors, which may introduce bias into the results. On the other hand, the factors considered in the matching process are relatively limited, such as not including the patient’s economic status, which could also influence the results.

The innovative aspect of our study is the exploration of TDM’s role in prophylactic antifungal treatment with posaconazole. We found that TDM contributes to optimizing treatment regimens, enhancing therapeutic outcomes, and reducing economic burdens on patients. Additionally, we utilized the PSM method to minimize selection bias, thus enhancing the reliability of our results. Our findings offer a new perspective for the rational use of posaconazole in clinical practice, expected to further improve treatment efficacy and patient survival rates.

## 5 Conclusion

In this study, children with hematopoietic stem cell transplantation who received posaconazole to prevent fungal infection were selected as the research objects, and the correlation between posaconazole TDM or not in the real world and clinical outcome was investigated. The results showed that compared with the non-TDM group, the TDM group could significantly increase the effective rate of fungal prevention, reduce the hospitalization cost and drug expenses of patients. This indicates that TDM can not only ensure the effectiveness of posaconazole but also reduce the economic burden on patients. However, in terms of the incidence of adverse events, there was no statistical difference between the two groups. Based on these research results, we recommend routine TDM for HSCT children who receive posaconazole for fungal prophylaxis to optimize drug efficacy and economy.

## Data Availability

The raw data supporting the conclusions of this article will be made available by the authors, without undue reservation.
